# Effects of Japan tallow on gut microbiota in type 2 diabetic mice

**DOI:** 10.1186/s12986-026-01079-3

**Published:** 2026-01-11

**Authors:** Kaiya Xie, Yuqing Zhang, Lucas Ji Zong Yu, Xin Yu, Jiangbo He, Yingzhen Su

**Affiliations:** 1 School of Medicine, Kunming University, Kunming, 650214 Yunnan China; 2 Yunnan Ruihao lifescience technology Ltd, Kunming, 650200 Yunnan China; 3 Uppsala Musikklasser, Prästgatan 13, Uppsala, 75228 Sweden

**Keywords:** T2DM, Japan tallow, Gut microbiota, Metabolism

## Abstract

**Background:**

Black Japan tallow (BJT) and white Japan tallow (WJT), collectively referred to as Japan tallow (JT), are traditional edible oils widely consumed in the Nujiang region of Yunnan, China. JT has been traditionally used for its potential health benefits, including anti-diabetic effects. However, scientific evidence supporting these claims is limited, and the underlying mechanisms remain unclear. This study aimed to evaluate the anti-diabetic effects of JT and to explore whether its action involves modulation of the gut microbiota.

**Methods:**

The lipidomic profile of JT was first characterized. Thirty C57BL/6J mice were randomly divided into five groups (*n* = 6): control group (ND), T2DM group (T2DM), dietary intervention group (CTRL), metformin treatment group (MET), and JT intervention group (JT). T2DM was induced by a high-fat diet (HFD) combined with low-dose streptozotocin (STZ) injections in all groups except ND. Body weight, food intake and fasting blood glucose (FBG) were monitored weekly. Metabolic responses were assessed using oral glucose tolerance test (OGTT) and insulin tolerance test (ITT). After four weeks, fecal samples were collected for 16 S rDNA sequencing, and tissue samples from the colorectum, liver, and pancreas were harvested for histological analysis.

**Results:**

Lipidomic analysis identified 230 distinct lipid molecules in JT. BJT showed higher abundances of unsaturated ceramide (Cer t18:1_22:1) and OAHFA (42:7). JT intervention significantly improved glycemic control, enhanced insulin sensitivity, and ameliorated tissue damage in the colorectum, liver, and pancreas of T2DM mice. Furthermore, 16 S rDNA sequencing indicated that JT intervention restored gut microbiota balance, shifting its composition toward a healthy state.

**Conclusion:**

JT exhibits significant anti-diabetic effects in a mouse model of T2DM, likely mediated through the restoration of gut microbiota homeostasis and improvement of metabolic parameters. These findings provide experimental support for the traditional use of JT and form a basis for future studies on its translational potential.

**Graphical Abstract:**

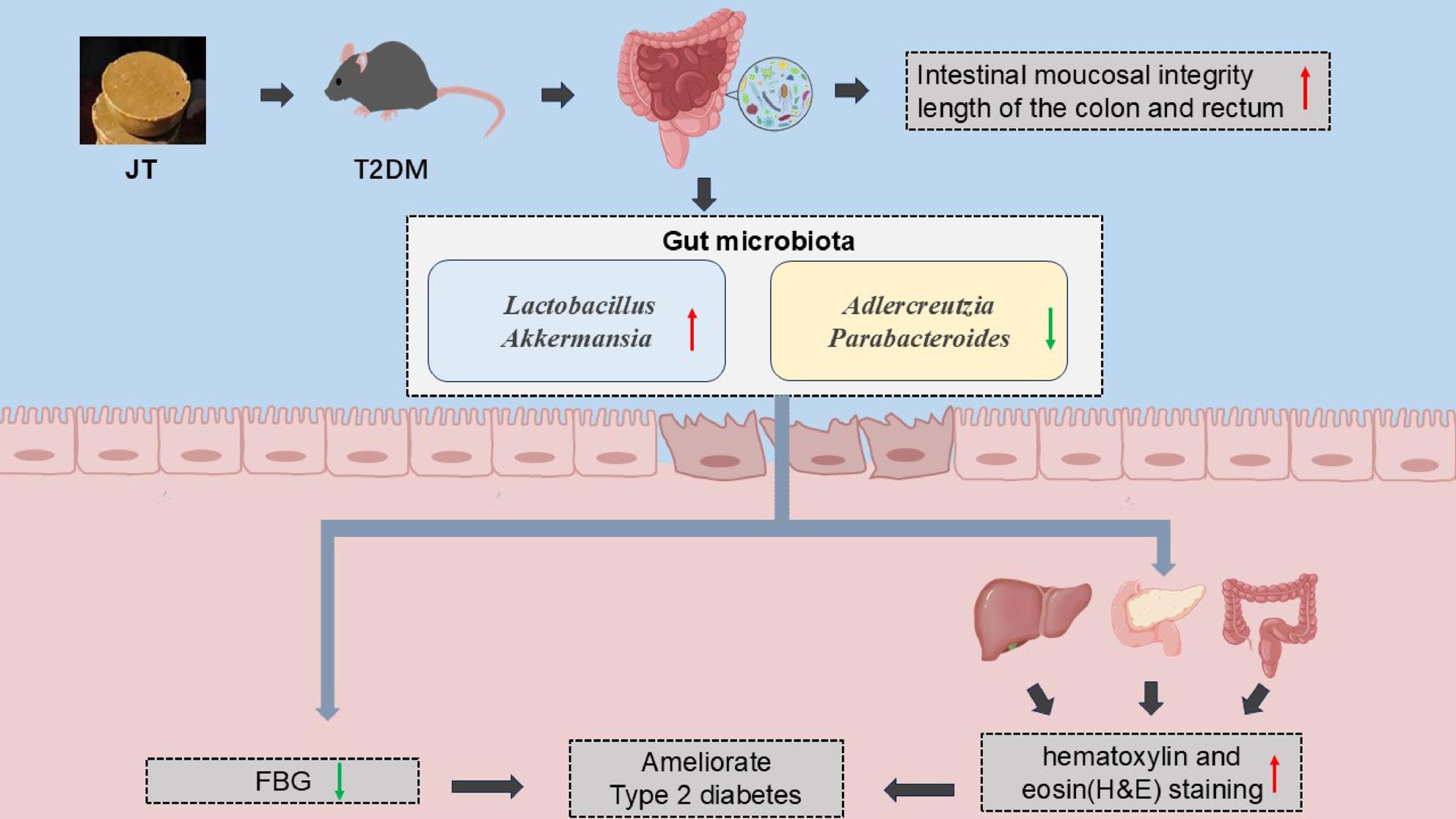

**Supplementary Information:**

The online version contains supplementary material available at 10.1186/s12986-026-01079-3.

## Introduction

Global shifts in societal structures and lifestyle patterns have coincided with a marked increase in diabetes prevalence, with type 2 diabetes mellitus (T2DM) constituting over 90% of all diagnosed cases. As a prevalent chronic metabolic disorder, T2DM is principally defined by impaired pancreatic β-cell function and peripheral insulin resistance, culminating in relative insulin deficiency [1]. T2DM is commonly associated with harmful complications, such as retinopathy, nephropathy, cardiovascular disease, and neuropathy [2–4]. It not only brings physical and mental suffering to patients, but also leads to significant medical economic burden for either society and families. According to the recent reports, the 11th edition of the International Diabetes Federation’s diabetes map showed that the number of people affected by diabetes has reached 537 million in 2021 around the world, and these numbers will rise to 783 million by 2045 [5]. Hence, effective prevention and treatment are essential for type 2 diabetes.

The escalating worldwide prevalence of T2DM has been linked to numerous risk factors, including the obesity pandemic, sedentary behaviors, and demographic aging. Growing scientific evidence suggests that intestinal microbiota imbalance may participate in the development of diabetes-related complications, whereas preserving microbial equilibrium appears essential for metabolic homeostasis [6–8]. Current pharmaceutical approaches for T2DM control, including metformin and α-glucosidase inhibitors (e.g., acarbose, miglitol, voglibose), though clinically efficacious, often present notable side effects and impose significant economic costs [9, 10]. For example, common gastrointestinal symptoms such as diarrhea may evolve into more serious hepatic impairments. These constraints of conventional treatments have stimulated increased interest in identifying safer, economically viable alternatives from natural sources capable of modulating gut microbiota in diabetic individuals.

Throughout history, medicinal plants have maintained important roles in traditional therapeutic systems across diverse cultures. In modern scientific inquiry, plant-derived compounds are receiving heightened attention for their potential application in metabolic disorder management. The sustained global utilization of phytotherapy, employed by an estimated 3.5–4.5 billion people worldwide [11], may be explained by the multifaceted bioactive components present in botanicals that demonstrate pharmacological activities. This enduring tradition of plant-based healing highlights its promise as a valuable source for developing innovative antidiabetic therapeutics.

Among botanicals with documented health benefits, Rhus verniciflua (commonly referred to as sumac) yields a natural wax that solidifies at ambient temperature due to its elevated melting point, hence termed “Japan tallow” (JT). This substance is classified into two variants: black Japan tallow (BJT) and white Japan tallow (WJT). BJT, originating from the Anacardiaceae family, has historical applications in diabetes management [12]. The wax is obtained from the seed coating of the sumac tree, with BJT extracted from unripe seeds at higher elevations and WJT from mature seeds at lower altitudes. Traditional medical practice attributes superior quality, enhanced nutritional value, and greater therapeutic potency to BJT [13]. Existing studies have confirmed that Japan tallow exhibits anti-inflammatory and antioxidant properties [12, 14, 15]. yet its mechanistic actions—particularly regarding gut microbiota modulation and potential antidiabetic benefits—remain inadequately investigated. This research seeks to evaluate the therapeutic efficacy of JT in a murine T2DM model, emphasizing its impact on intestinal microbial community regulation. The findings may provide experimental support for the development of JT-based interventions in diabetes management.

## Materials and methods

### Samples collection

The black JT (BJT) used in this study was sourced from Nujiang Autonomous Prefecture, Yunnan Province, China. This specific batch was selected based on the well-documented profile of BJT as a lipid-rich substance, historically characterized by oleic, palmitic, and stearic acids as its major fatty components. The JT was produced by local artisans using a traditional physical pressing method, which involved roasting the seeds of the lacquer tree (*Rhus verniciflua*), mechanically crushing them with a stone mill, and extracting the oil using a wooden lever-press without chemical solvents. The batch was purchased within three months of production and stored in our laboratory at 4℃ under dark, dry conditions until use.

### Reagents

Streptozotocin (STZ, S8050), a hematoxylin-eosin (HE) staining kit (G1120), and sodium citrate buffer (SSC, C1013) were purchased from Solarbio Biotechnology (Beijing, China). Metformin (MET, S30880) was purchased from Yuanye Biotechnology (Shanghai, China).

### Animals

A total of 30 specific pathogen-free (SPF) male C57BL/6J mice (5 weeks old, body weight: 18–20 g) were purchased from Hunan Silaike Jingda Laboratory Animal Co., Ltd. (Production License No.: SCXK (Xiang) 2019-0004). The mice were housed in the animal laboratory of Kunming University under controlled conditions (temperature: 20–26 ℃; relative humidity: 50–60%) with free access to water. All experimental procedures were approved by the Animal Welfare and Ethics Committee of Kunming University (Approval No.: KMU2025070).

### Animal modeling and experimental design

**Fig. 1:**
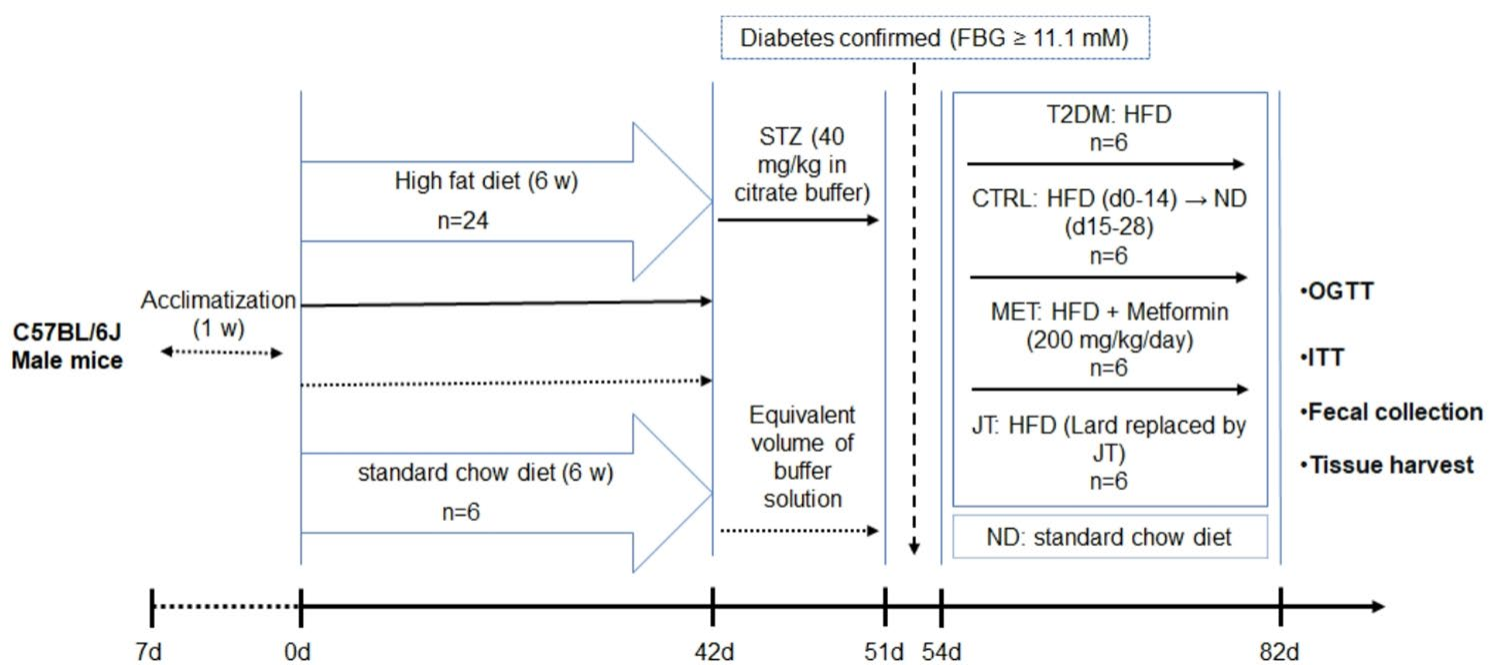
Schematic timeline of the experimental design

Thirty male C57BL/6J mice were acclimatized for 7 days and then randomly assigned to two groups based on fasting blood glucose levels: a normal diet control group (ND, *n* = 6) fed a standard chow, and a high-fat diet (HFD) group (*n* = 24). The HFD group received a custom high-fat diet with the following composition (per kilogram of diet): 400 g casein, 6 g cystine, 250 g maltodextrin, 145.6 g sucrose, 100 g cellulose, 470 g lard, 50 g soybean oil, 100 g commercial mineral mix, 2 g commercial vitamin mix, and 1 g choline bitartrate. This formulation provided approximately 60% of calories from fat. Following 6 weeks of HFD feeding, the mice were administered three intraperitoneal injections of STZ (40 mg/kg in citrate buffer) at 3-day intervals to induce gradual beta-cell dysfunction. Diabetes induction was confirmed 72 h after the final injection through tail vein blood glucose measurement, with fasting blood glucose ≥ 11.1 mmol/L serving as the criterion for successful T2DM model establishment [16]. The diabetic mice were subsequently randomized into four treatment groups (*n* = 6/group) for a 4-week intervention: T2DM group (T2DM; *n* = 6) maintained on high-fat diet; dietary intervention group (CTRL; *n* = 6) receiving HFD for days 0–14 followed by standard diet for days 15–28; metformin treatment group (MET; *n* = 6) administered HFD plus 200 mg/kg/day metformin via oral gavage; and JT intervention group (JT; *n* = 6) fed a modified HFD in which lard was iso-proportionally replaced by JT (470 g/kg). All other ingredients remained unchanged. The detailed experimental timeline is schematically illustrated in Fig. 1

### Lipidomic study

For lipid extraction, we employed a biphasic chloroform-methanol system. Approximately 50–100 mg of tissue was homogenized in 750 µL of chilled (−20℃) chloroform: methanol (2:1, v/v) using a 2 mL tube, followed by 30 s vortexing and 40 min ice incubation. Phase separation was induced by adding 190 µL H₂O with additional vortexing (30 s) and 10 min on ice. After centrifugation (12,000 rpm, 5 min, RT), 300 µL of the lower organic phase was collected. The remaining aqueous phase was re-extracted with 500 µL fresh solvent mixture, vortexed, and centrifuged again, yielding another 400 µL organic phase. Combined extracts were vacuum-dried, reconstituted in 200 µL isopropanol, and filtered (0.22 μm) prior to LC-MS analysis. For limited samples, all volumes were scaled down proportionally.

### Metabolic parameter measurements

Body weight and fasting blood glucose (FBG) were measured at baseline (Day 0) and on Days 3, 7, 15, 18, 21, and 28 of the intervention. FBG measurements were performed after a 12-hour overnight fast (from 8:00 PM to 8:00 AM), with free access to water. A blood sample was obtained via tail vein puncture using a sterile lancet, and glucose concentration was immediately determined using a Contour TS blood glucose monitoring system. All sampling was conducted between 8:00 AM and 10:00 AM to minimize diurnal variation.

Food intake was assessed weekly. To avoid the acute stress of blood sampling, measurements were conducted over 3–4 consecutive days within each week, scheduled on days without FBG tests. The average daily food intake per mouse was calculated from the difference between the food provided and the remaining food per cage.

### Oral glucose tolerance test and insulin tolerance test

After 4 weeks of drug treatment, the mice were subjected to the oral glucose tolerance test (OGTT), which was performed after a 12 h fasting period before the experiment and after oral gavage of glucose solution at a dose of 2 g/kg. Blood glucose concentration was measured at 0,30,60,90 and 120 min, and the area under the curve (AUC) was calculated. insulin tolerance test (ITT) was performed. Mice were fasted for 4 h and intraperitoneally injected with insulin (0.4 units/kg). Blood glucose concentration was measured at 0,15,30,45 and 60 min, and the AUC was calculated.

### Histopathological analysis

The liver, colorectal tissues, and pancreas were collected and fixed in 4% buffered formaldehyde, then embedded in paraffin and sectioned at 4 μm for histological analysis. Tissue sections were stained with HE following standardized protocols. Histopathological alterations in the liver, colorectal region, and pancreas were subsequently examined under polarized light microscopy for detailed evaluation [17–19].

### 16S rDNA gene sequencing

Microbial community analysis was performed on 200 mg fecal samples obtained from experimental mice. After implementing optimized DNA extraction protocols, DNA quantity and quality were evaluated through spectrophotometric measurement (Nanodrop ND-1000) and electrophoretic separation on 1.2% agarose gels. The complete 16 S rDNA sequencing workflow and subsequent bioinformatics analyses were conducted by Suzhou Panomik Biomedical Technology Co., Ltd. Following bacterial DNA isolation with the Solebo D2700 extraction kit, the V3-V4 hypervariable regions were PCR-amplified using indexed universal primers (338 F: 5’-ACTCCTACGGGAGCAGCAG-3’; 806R: 5’-GGACTACHVGGGTWTCTA-3’). Amplification success was confirmed by 2% agarose gel electrophoresis before library preparation and sequencing on the Illumina NovaSeq PE250 platform (Illumina, San Diego, CA).

The bioinformatics pipeline included: (1) quality-based trimming of raw reads using Trimmomatic (v0.39), (2) paired-end read assembly with FLASH (v1.2.11), and (3) chimera detection and removal employing the UCHIME algorithm. Processed sequences were then clustered into operational taxonomic units (OTUs) at 97% nucleotide similarity threshold via UPARSE (v11) and subsequently classified taxonomically. This comprehensive approach enabled detailed profiling of both abundant and low-frequency microbial taxa present in the fecal specimens.

### Statistical analysis

Statistical analyses were performed using GraphPad Prism 9.5 (GraphPad Software Inc., USA). Depending on data characteristics, we employed either one-way or multifactorial ANOVA after confirming normality assumptions, while non-normally distributed datasets were analyzed using Kruskal-Wallis rank sum tests. Throughout our analyses, a two-tailed p-value threshold of 0.05 served as the criterion for establishing statistical significance, with all post-hoc comparisons appropriately adjusted for multiple testing.

## Results

### Overall lipidomics analysis of JT

**Fig. 2:**
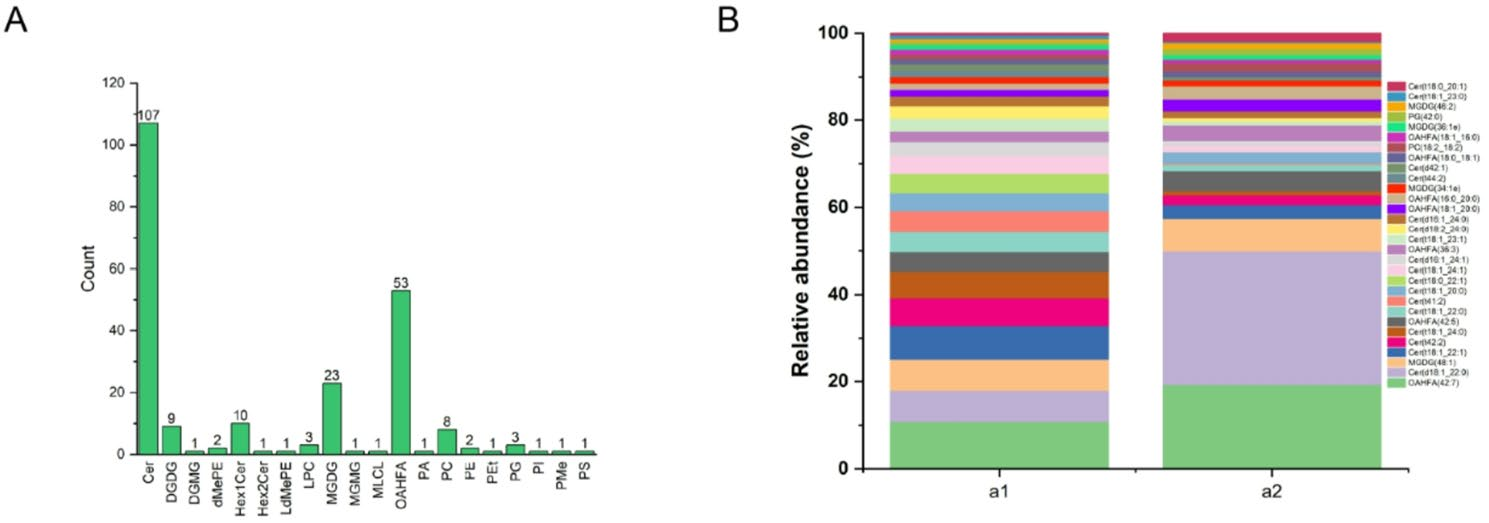
Lipid composition analysis of BJT and WJT. (**A**) Distribution of each lipid subclass identified. (**B**) lipid subclasses in JT. a1, BJT; a2, WJT

Lipids are a large group of organic compounds that are insoluble in water but soluble in organic solvents and are also called lipids. These include phospholipids, sphingolipids, neutral lipids and glycolipids, all of which are important components of lipids and play a key role in the structure and function of organisms. In this study, 230 lipid molecules were detected, including 107 Ceramides (Cer), 9 Digalactosyldiacylglycerol (DGDG), 1 Digalactosylmonoacylglycerol (DGMG), 2 dimethylphosphatidylethanolamine (dMePE), 10 Simple Glc series (Hex1Cer), 1 Simple Glc series (Hex2Cer), 1 lysodimethylphosphatidylethanolamine (LdMePE), 3 lysophosphatidylcholine (LPC), 23 Monogalactosyldiacylglycerol (MGDG), 1 Monogalactosylmonoacylglycerol (MGMG), 1 Cardiolipin (MLCL), 53 OAcyl-(gamma-hydroxy)FA (OAHFA), 1 phosphatidic acid (PA), 8 phosphatidylcholine (PC), 2 phosphatidylethanolaines (PE), 1 phosphatidylethanol (PEt), 3 phosphatidylglycerol (PG), 1 phosphatidylinositiols (PI), 1 phosphatidylmethanol (PMe), 1 phosphatidylserines (PS) (Fig. 2A). As shown in Fig. 2B, lipids were classified into 3 categories. The proportions of the different molecules and lipid classes in JT were analyzed according to their relative contents. Among the lipid subclasses, regarding black Japan tallow (a1) and white Japan tallow (a2), the top components were OAHFA (42:7) (10.8%), Cer (t18:1_22:1) (32.9%) and OAHFA (42:7) (19.3%), Cer (d18:1_22:0) (49.7%).

### Effects of JT on food intake, glucose and weight levels on T2DM mice

**Fig. 3:**
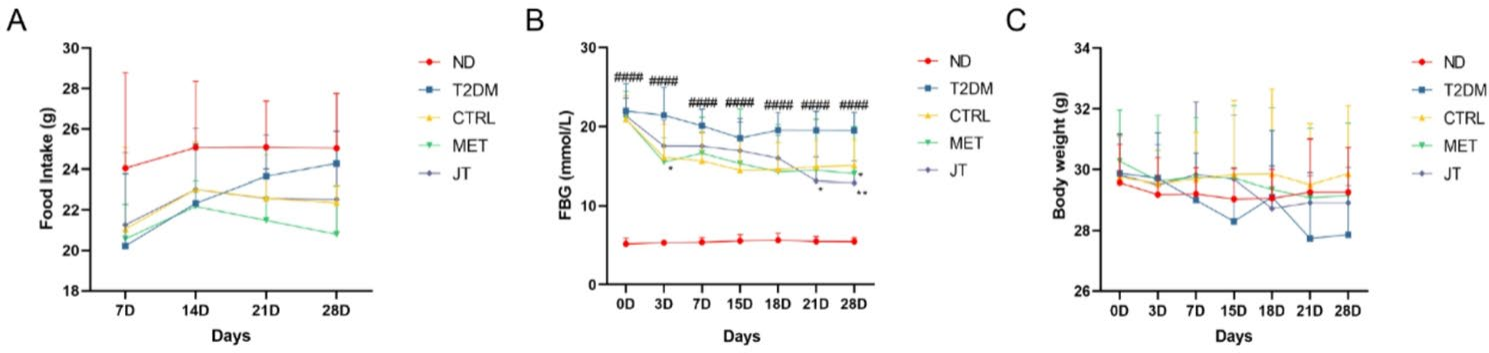
JT treatment reduces fasting blood glucose without significantly altering body weight or food intake in T2DM mice. (**A**) Weekly food intake in mice. (**B**) BG was controlled after JT treatment. (**C**) body weight of mice in different groups. Data were expressed as the mean ± SD. *n* = 6. ####*P* < 0.0001 vs. ND, *P < 0.05 vs. T2DM, **P < 0.01 vs. T2DM

In order to study the antidiabetic effects of JT, T2DM mice were oral feed with JT for 28 days. Food intake among the groups showed no significant difference (Fig. 3A). Significant changes in fasting blood glucose (FBG) in control and experimental groups were detected (Fig. 3B). Significant decrease of FBG levels were observed under both JT and MET intervention in T2DM mice. In the JT group, the blood glucose levels of T2DM mice were significantly decreased compared to T2DM mice without intervention. Metformin intervention was used as the positive control. JT intervention showed no significant effects on the body weight (Fig. 3C).

### JT intervention improved both glucose and insulin tolerance in T2DM mice

**Fig. 4:**
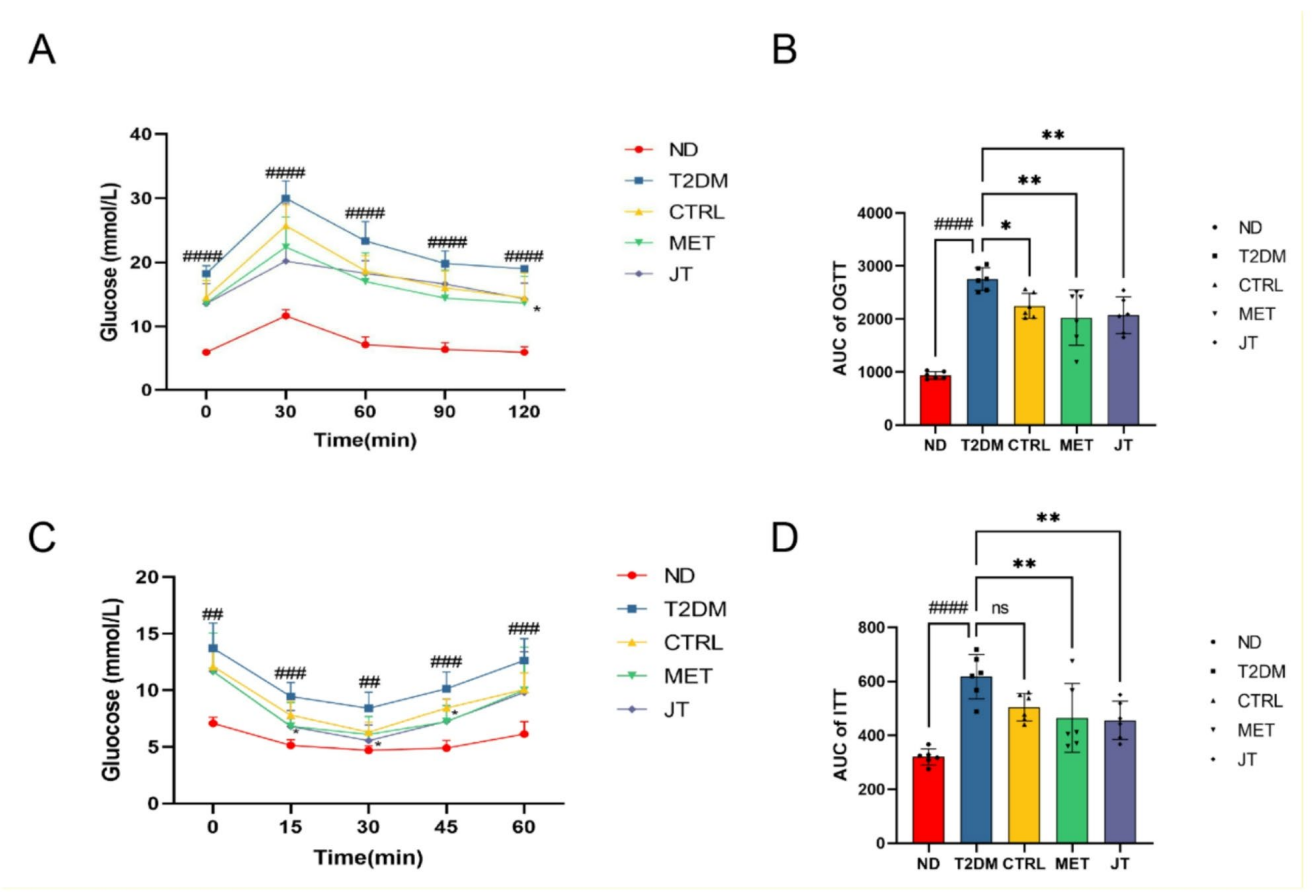
JT improved glucose tolerance and insulin resistance in T2DM mice. (**A**) OGTT. (**B**) AUC corresponding to OGTT. (**C**) ITT. (**D**) AUC corresponding to the ITT. Data were expressed as the mean ± SD. n = 6. ##*P* < 0.01 vs. ND, ###*P* < 0.001 vs. ND, ####*P* < 0.0001 vs. ND, *P < 0.05 vs. T2DM, **P < 0.01 vs. T2DM, ns = no significant difference

Oral glucose tolerance test (OGTT) and insulin tolerance test (ITT) are important experimental parameters to evaluate diabetes. Changes in blood glucose and insulin levels are generally used to show the utilization of glucose and then indicating whether the islet dysfunction or healthy. To investigate the glucose tolerance and insulin sensitivity of mice, after 4 weeks of drug intervention, we performed OGTT and ITT on all group mice (Fig. 4). Compared to the ND group, the T2DM group mice showed significantly increased insulin resistance. Compared to the T2DM group, MET and JT group mice were protected from T2DM-induced systemic glucose intolerance and insulin resistance (Fig. 4A-D).

### JT intervention inhibited the shortened colon and rectum in T2DM mice

**Fig. 5:**
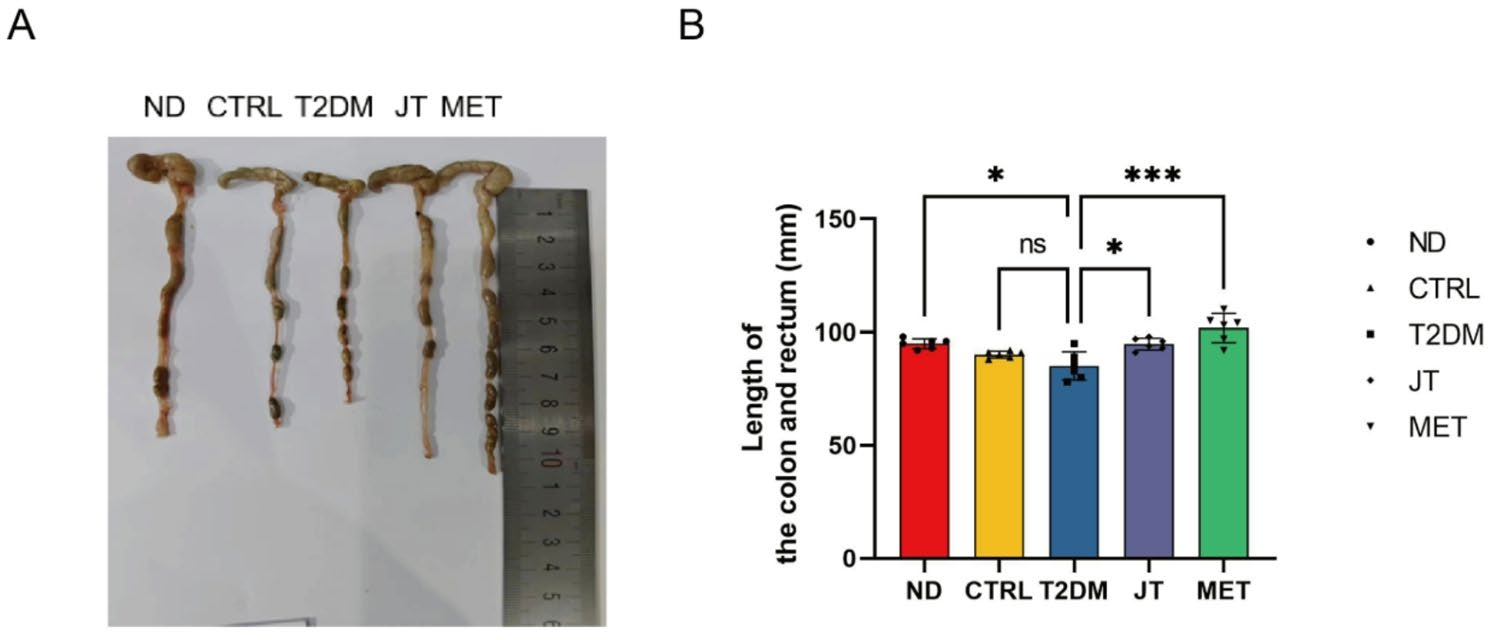
JT significantly prevent the shorten of colon and rectum in type 2 diabetic mice. (**A**) Representative gross photographs of the colorectum from each group. (**B**) Quantification of colorectal lengths in each group. Data were expressed as the mean ± SD. n =6. *P < 0.05; ***P < 0.001; ns = no significant difference

Figure 5 demonstrates that the T2DM group exhibited significantly shorter colon and rectum lengths compared to the ND group. However, treatment with JT and MET significantly restored colon and rectum lengths relative to the T2DM group. These findings suggest that JT supplementation may effectively ameliorate T2DM-associated colorectal morphological alterations.

### JT recovers the harmful effects on colorectum, liver and pancreas in T2DM

**Fig. 6:**
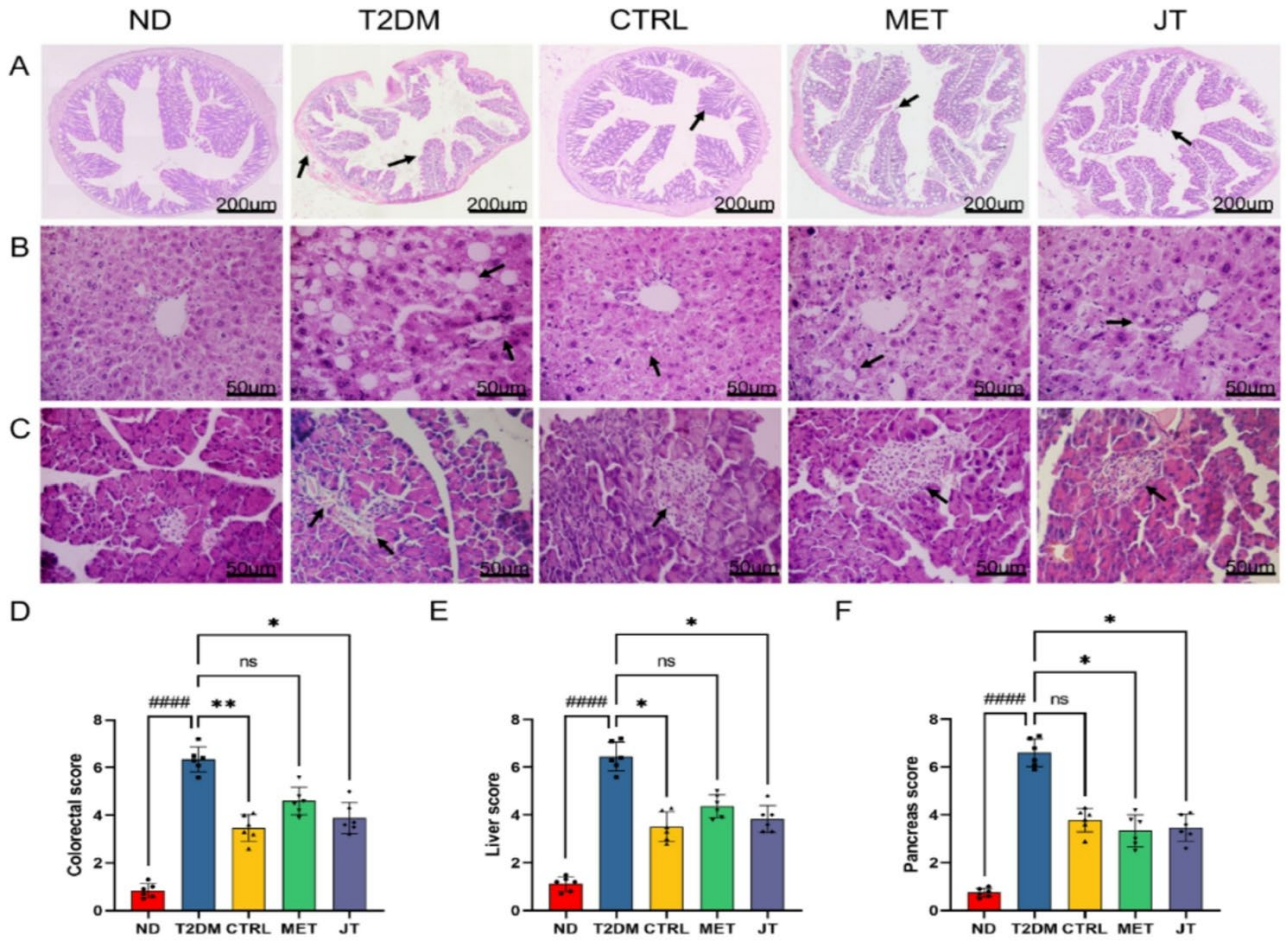
HE staining indicated that the histopathological changes of colorectum, liver, and pancreas were reduced in T2DM model mice after JT treatment. (**A**) Morphological changes of colorectal tissue. (**B**) Morphological changes of liver tissue. (**C**) Morphological changes of pancreas. (**D**) Histopathological score of the colorectal tissue. (**E**) Histopathological score of the liver tissue. (**F**) Histopathological score of the pancreatic tissue. Data were expressed as the mean ± SD. *n* = 6. ####*P* < 0.0001 vs. ND, *P < 0.05 vs. T2DM, **P < 0.01 vs. T2DM, ns = no significant difference

In order to investigate the effects of JT on histomorphological level, HE staining of colorectum were tested as shown in Fig. 6 In T2DM mice, colorectal submucosa edema and mucosal damage were observed, accompanied by the extensive loss of goblet cells. In contrast, mice in the MET, CTRL, and JT groups exhibited an absence of submucosal edema, only a minimal loss of goblet cells, along with a marked reduction in the severity of mucosal and crypt damage compared to the T2DM group (Fig. 6A, D).

HE staining of liver revealed that the hepatic cords of mice were disordered in T2DM group, with more inflammatory cell infiltration and a large number of hepatocytes vacuolation and steatosis. The hepatic cords of each treatment group were neatly arranged after treatment, with a small amount of inflammatory cell infiltration and scattered hepatocyte vacuolation and steatosis seen (Fig. 6B, E).

HE staining of pancreatic tissue showed that the structure of islets in the ND group was regular round, uniform in size, clear in edge, saturated in shape, and closely arranged, and no vacuoles or obvious pathological changes were observed. In T2DM group, the islet morphology was irregular, the distribution was uneven, the structure was disordered, the boundary was fuzzy, and the cells were deformed. Compared with T2DM mice, the islet structure of MET, CTRL and JT mice showed regular shape, clear boundary, significantly increased cell number, no obvious necrosis, and improved morphology and structure (Fig. 6C, F).

### Shannon diversity and PCoA reveal community differences

**Fig. 7:**
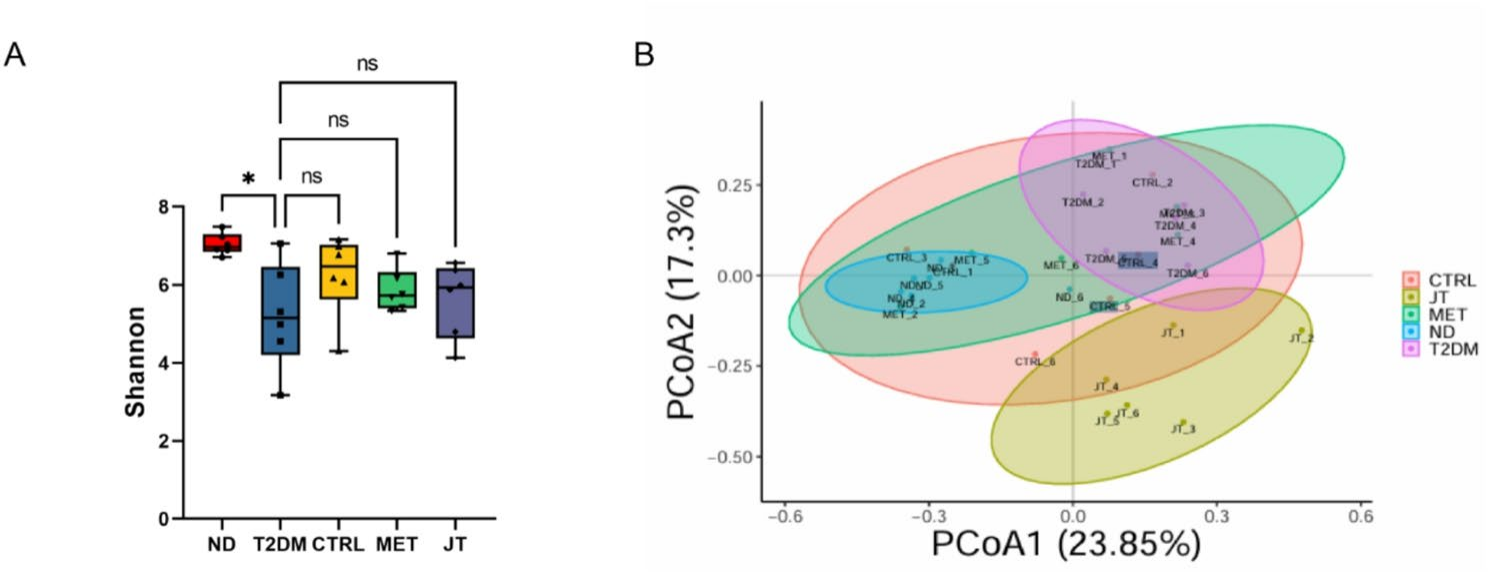
Microbial community diversity and composition. (**A**) Shannon index reflecting alpha diversity. (**B**) PCoA plot based on Bray-Curtis distances showing beta diversity. Data were expressed as the mean ± SD. *n* = 6. *P < 0.05; ns = no significant difference

The alpha diversity, as measured by the Shannon index, revealed a significant decrease (*p* < 0.05) in the T2DM group compared to the ND group ((Fig. 7A). Although no statistically significant differences were observed between the T2DM group and the CTRL, MET, or JT groups, all three intervention groups exhibited a trend toward increased microbial diversity relative to the T2DM group.

Principal coordinates analysis (PCoA) of beta diversity demonstrated distinct clustering patterns among the five groups ((Fig. 7B), indicating compositional differences in gut microbiota structure. The separation between groups suggests that both T2DM and therapeutic interventions (CTRL, MET, and JT) influenced microbial community composition.

### The structure of the intestinal microflora was altered under JT treatment

**Fig. 8:**
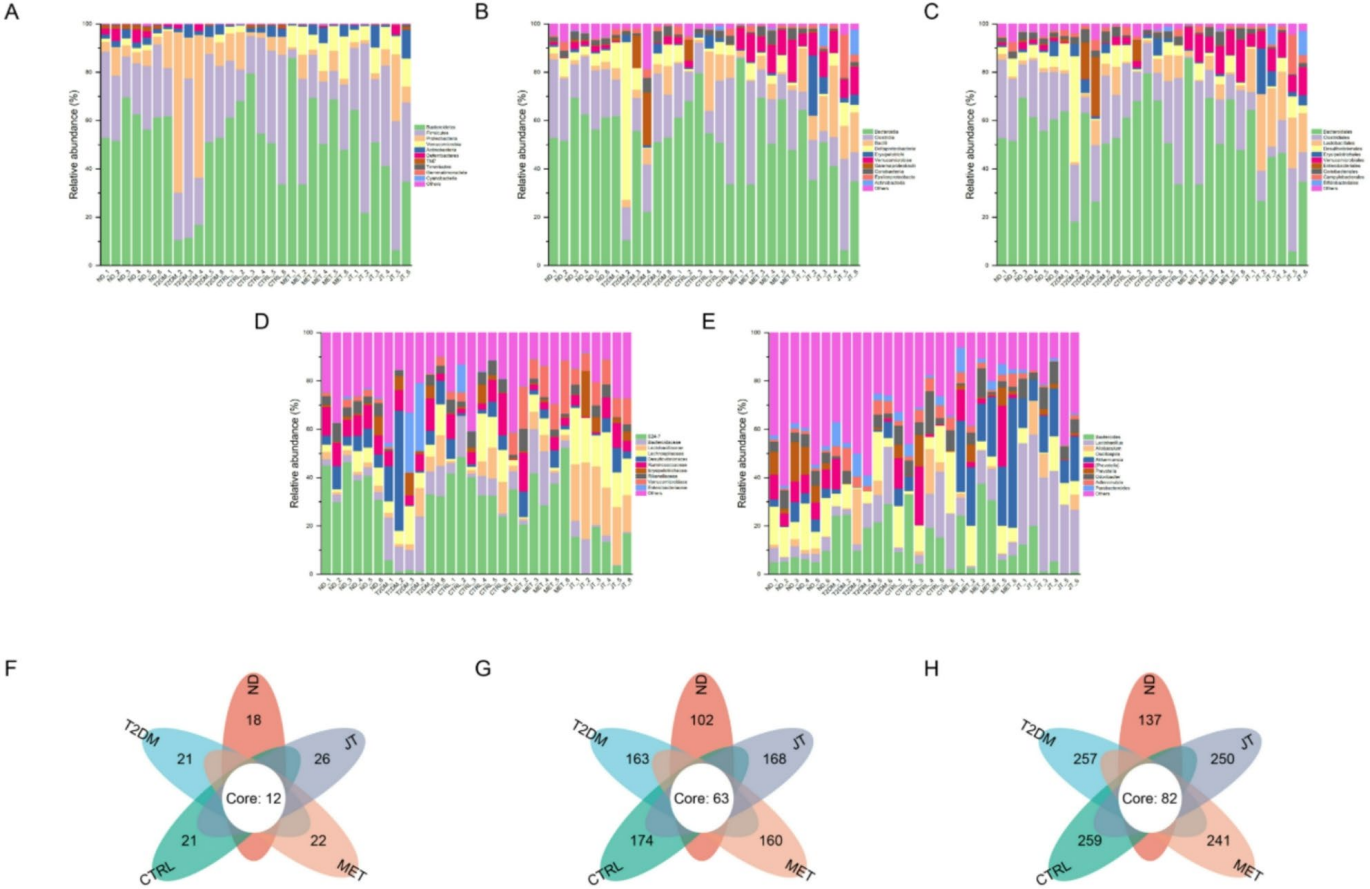
Gut microbiota composition and treatment-specific signatures. (**A-E**) Relative abundance of the top 10 taxa at the (**A**) phylum, (**B**) class, (**C**) order, (**D**) family, and (**E**) genus levels. (**F-H**) Treatment-specific microbial signatures revealed by Venn analysis at the (**F**) phylum, (**G**) family, and (**H**) genus levels. Venn diagrams show the counts of unique and shared operational taxonomic units (OTUs) among the ND, T2DM, CTRL, MET and JT groups. The number in each section indicates the count of OTUs

Microbial community analysis revealed distinct taxonomic shifts associated with T2DM progression and JT intervention. At the phylum level, Firmicutes and Bacteroidota dominated the gut microbiota composition across all groups (Fig. 8A-E). The T2DM group exhibited characteristic dysbiosis, showing an increase in Proteobacteria and reduction in Verrucomicrobiota compared to CTRL controls. JT administration mitigated these changes, normalizing the Proteobacteria abundance toward control levels. Venn analysis demonstrated treatment-specific microbial signatures, with JT-treated mice harboring 26 unique phylum-level OTUs versus 21 in T2DM controls (Fig. 8F). This pattern extended to finer taxonomic classifications, where JT maintained 168 family-level and 250 genus-level OTUs (Fig. 8G, H), representing intermediate diversity between healthy and diabetic states. The observed microbial restructuring, particularly the JT-mediated regulation of Proteobacteria and Verrucomicrobiota, aligns with the compound’s glucose-lowering effects observed in metabolic parameters.

### JT intervention modified the gut microbial composition at the genus level in T2DM mice

**Fig. 9:**
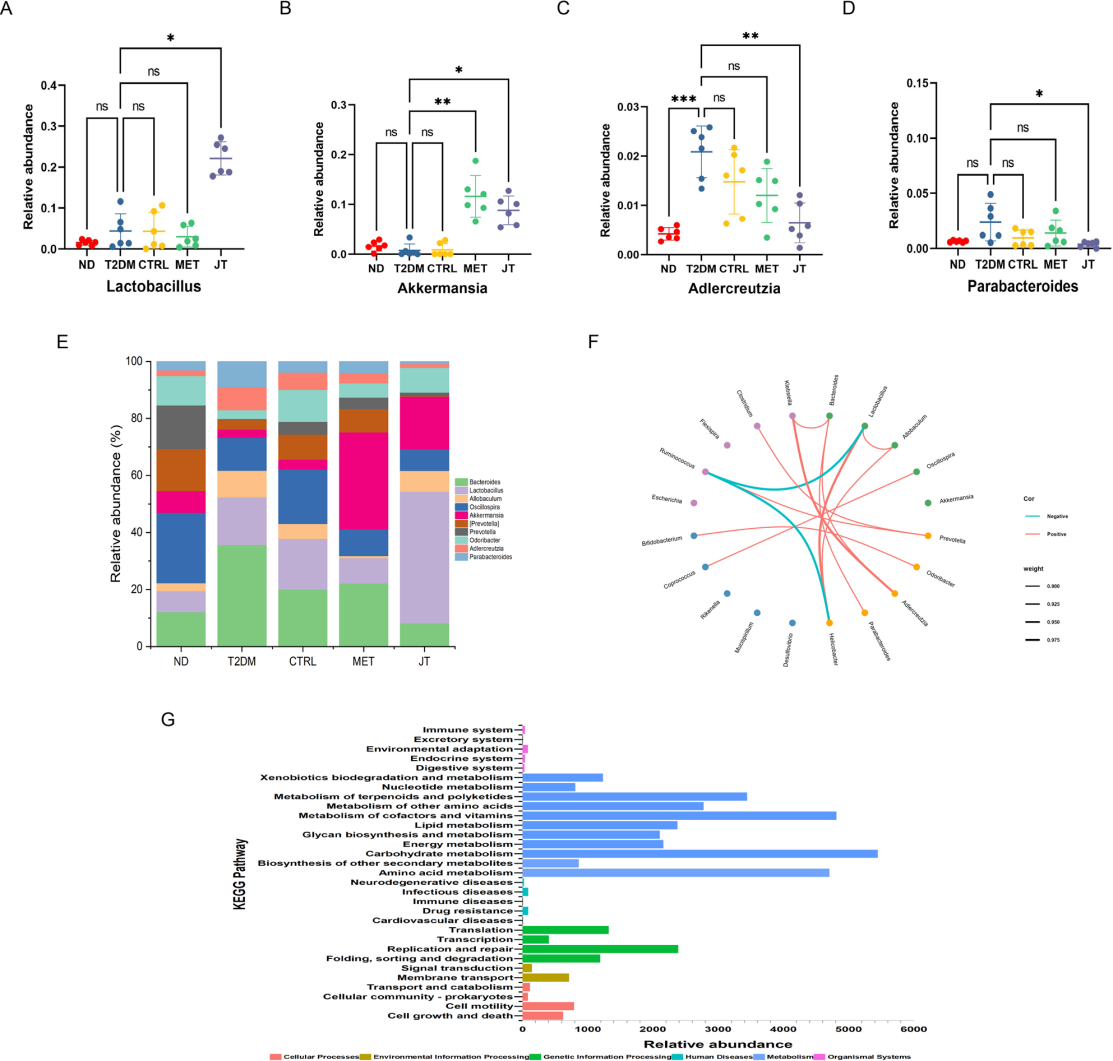
JT intervention could recover the microbiota structure of type 2 diabetes mellitus mice. (**A-D**) Differences in the top 10 abundances under JT intervention; (**E**) Genus-level composition showing percentage distribution of major taxa; (**F**) Co-occurrence network analysis of gut microbiota at the genus level. The red lines (positive correlations) and the blue lines (negative correlations). (**G**) KEGG analysis of relative abundance of metabolic pathways. The magnitude of the correlation is expressed by the thick ness of the line. n=6. *P < 0.05; **P < 0.01; ***P < 0.001; ns = no significant difference

Our comprehensive analysis revealed significant JT-induced alterations in gut microbiota composition and function at multiple levels. At the genus level, JT intervention markedly increased the relative abundance of beneficial taxa including Lactobacillus and Akkermansia, while reducing potential pathobionts such as Adlercreutzia and Parabacteroides (Fig. 9A-D). Taxonomic profiling confirmed Lactobacillus as a dominant genus in JT-treated mice, accompanied by significant suppression of Parabacteroides (Fig. 9E), suggesting a direct association between these microbial shifts and JT’s anti-diabetic effects.

The co-occurrence network analysis provided deeper insights into microbial ecological relationships (Fig. 9F). JT treatment not only promoted the expansion of beneficial Lactobacillus but also enhanced its positive interactions with Helicobacter, while suppressing diabetes-associated Ruminococcus. Notably, Akkermansia, a genus strongly associated with metabolic health, emerged as an independent network node following JT intervention. Concurrently, potential pathogenic taxa including Parabacteroides and Klebsiella exhibited reduced network connectivity, maintaining only limited interactions with Bacteroides and Adlercreutzia.

These structural changes were further analyzed for their predicted functional implications using KEGG pathway analysis based on 16 S rDNA gene sequences. The analysis indicated significant enrichment in multiple metabolic pathways critical for glucose homeostasis, including carbohydrate metabolism, amino acid metabolism, and lipid metabolism (Fig. 9G). The coordinated modulation of microbial composition and their predicted metabolic profiles supports a hypothesis that JT ameliorates T2DM through a dual mechanism: restructuring gut microbial communities by promoting beneficial taxa and suppressing harmful ones, and enhancing microbial metabolic functions that may collectively contribute to improved host metabolic status.

The convergence of taxonomic changes (increased Lactobacillus/Akkermansia), ecological network remodeling (enhanced beneficial microbial cooperations), and metabolic pathway enrichment provides compelling evidence for JT’s multi-faceted anti-diabetic mechanisms through gut microbiota modulation.

## Discussion

Mounting research reveals that perturbations in lipid homeostasis play a central role in T2DM development and progression. While the pathogenic effects of certain lipid classes are well-documented, recent investigations provide deeper mechanistic understanding of how individual lipid molecules influence disease pathways. Notably, our lipidomic characterization of JT varieties uncovered significant compositional differences, with the black variant exhibiting elevated levels of potentially beneficial lipid species.

The BJT’s distinctive lipid profile features elevated levels of unsaturated ceramide (Cer t18:1/22:1) and OAHFA (42:7). These findings hold therapeutic potential, as experimental studies demonstrate that unsaturated ceramides (e.g., C24:1) mitigate the metabolic toxicity of saturated ceramides (e.g., C16:0) by avoiding deleterious effects on insulin signaling and mitochondrial function [20]. Additionally, OAHFAs (a subclass of fatty acid esters of hydroxyl fatty acids, FAHFAs) have been shown to enhance gut barrier integrity and reduce inflammation, which is frequently impaired in metabolic disorders such as diabetes [21]. Our data indicate that BJT intervention was associated with reduced expression of IL-1β, a pro-inflammatory cytokine (Supplementary Fig. 1). Previous studies have reported that BJT can increase OAHFA levels, and OAHFAs have been linked to anti-inflammatory effects [21]. Thus, the observed decrease in IL-1β could be consistent with a role for OAHFAs in mediating this response. Separately, IL-1β has been shown in other models to compromise intestinal epithelial integrity [22]. Taken together, these points allow us to propose a working hypothesis: BJT might influence gut health partially through an OAHFA-associated reduction in IL-1β, which could be relevant for maintaining barrier function. Testing this hypothesis definitively would require future studies that directly measure barrier parameters after BJT treatment.

The distinctive lipid profile of BJT indicates potential for T2DM intervention. Its dual bioactive components - the unsaturated ceramides that may help ameliorate metabolic dysfunction and the OAHFAs that could support epithelial integrity - suggest a multi-targeted approach to diabetes management. As a natural product, BJT merits consideration as a pharmacological alternative. To advance these findings, further research is needed, starting with mechanistic studies in appropriate disease models, followed by rigorous clinical evaluation to assess therapeutic efficacy and safety profiles.

Both JT and metformin (MET) significantly reduced fasting blood glucose levels in T2DM mice without affecting body weight, with food intake also showing no significant changes across groups (Figures. 3. A-C). This demonstrating comparable glucose-lowering efficacy while maintaining weight neutrality [23]—a clinically advantageous feature over other antidiabetic agents that induce weight gain. The improved glucose tolerance and insulin sensitivity in JT- and MET-treated mice (Fig. 4 A-D) suggest enhanced peripheral glucose uptake and preserved β-cell function, supported by restored islet morphology, aligning with gut microbiota-modulation strategies [24–26]. Previous studies have shown that certain unsaturated lipids can improve metabolic parameters in diabetes [27, 28]; while JT contains unsaturated ceramides (Cer t18:1_22:1) and OAHFA (42:7), further research is needed to establish their specific mechanisms in this model. These findings nevertheless position JT as a promising candidate for T2DM intervention, offering metabolic improvement without weight-related side effects.

Notably, JT demonstrated significant organ-protective properties beyond glycemic control. The observed prevention of diabetes-associated colorectal shortening — an indirect morphological indicator — alongside the restoration of mucosal architecture, is consistent with an overall improvement in intestinal structure. This structural improvement may be associated with enhanced gut barrier integrity, a factor implicated in modulating systemic metabolic homeostasis [29, 30]. The hepatoprotective effects, including reduced steatosis and inflammatory infiltration, are particularly valuable given the high prevalence of NAFLD in T2DM patients. The pancreatic islet protection further supports JT’s potential as a comprehensive antidiabetic intervention targeting multiple organ systems.

Our findings demonstrate that JT intervention induces measurable changes in gut microbiota composition in T2DM mice. The restoration of Proteobacteria levels toward control values is particularly significant, as elevated Proteobacteria abundance has been consistently associated with intestinal dysbiosis and metabolic dysfunction in both human and animal studies [31]. Similarly, the partial recovery of Verrucomicrobiota abundance following JT treatment suggests a modulatory effect on diabetes-associated dysbiosis, given that reduced Verrucomicrobiota levels have been linked to impaired gut barrier function in T2DM [32].

At finer taxonomic resolution, JT administration significantly increased Lactobacillus abundance while reducing Parabacteroides. These alterations correlate with the observed metabolic improvements and are consistent with previous reports showing that Lactobacillus species can improve glucose homeostasis through multiple mechanisms [33, 34], while Parabacteroides distasonis has been associated with metabolic dysfunction [35]. However, it should be noted that different Lactobacillus species may have distinct metabolic effects [36, 37], and our genus-level analysis cannot differentiate between potentially beneficial and neutral species.

The functional implications of these compositional changes are supported by enrichment of microbial metabolic pathways involved in carbohydrate and lipid metabolism. Similar pathway alterations have been reported in other studies of microbiota-targeted interventions for metabolic disorders [38]. Nevertheless, several important limitations must be acknowledged. First, while the increased Akkermansia genus abundance is noteworthy given its established association with metabolic health [39], our taxonomic resolution does not permit conclusions about specific species-level changes, particularly regarding Akkermansia muciniphila, which has been most extensively studied for its metabolic benefits [40–42].

The network analysis revealed altered inter-taxa relationships following JT treatment, particularly involving Lactobacillus interactions. These ecological changes mirror patterns observed with other microbiota-modulating therapies [43] and suggest that JT may influence microbial community stability. Combined with the observed taxonomic and functional alterations, these findings point to potential mechanisms by which JT may influence host metabolism. However, as demonstrated by recent studies using germ-free animal models [44], establishing causal relationships between specific microbial changes and metabolic outcomes requires additional experimental approaches.

Future studies employing metagenomic sequencing and targeted cultivation approaches will be essential to identify the specific microbial strains and functional genes responsible for JT’s metabolic effects. Additionally, fecal microbiota transplantation experiments could help determine whether the observed microbial changes are sufficient to mediate JT’s anti-diabetic effects [45, 46].

## Conclusions

In summary, our findings demonstrate that Japan tallow (JT) exerts potent anti-diabetic effects by improving glucose homeostasis and insulin sensitivity while protecting pancreatic islets, liver, and intestinal integrity in T2DM mice, likely mediated through its unique ceramide-rich lipid profile and beneficial modulation of gut microbiota (particularly Akkermansia and Lactobacillus), suggesting its potential as a novel multi-target therapeutic agent for diabetes management.

## Supplementary Information

Supplementary Material 1

## Data Availability

The data that support the findings of this study are available from the corresponding author upon reasonable request.

## Abbreviations

T2DM: Type 2 diabetes mellitus
JT: Japan tallow
STZ: Streptozotocin
SSC: Sodium citrate buffer
MET: Metformin
HE: Hematoxylin-eosin
SPF: Specific pathogen-free
FBG: Fasting blood glucose
HFD: High-fat diet
OGTT: Oral glucose tolerance test
ITT: Insulin tolerance test
AUC: Area under the curve

## References

[1] American Diabetes Association Professional Practice Committee. 2. Diagnosis and classification of diabetes: standards of care in diabetes-2024. Diabetes Care 2024;47 Suppl 1:S20-S42. 10.2337/dc24-S002 PMC10725812 38078589

[2] Guo W, Song Y, Sun Y, Du H, Cai Y, You Q, et al. Systemic immune-inflammation index is associated with diabetic kidney disease in type 2 diabetes mellitus patients: evidence from NHANES 2011–2018. Front Endocrinol (Lausanne). 2022;13:1071465. 36561561 10.3389/fendo.2022.1071465 PMC9763451

[3] Khalili F, Vaisi-Raygani A, Shakiba E, Kohsari M, Dehbani M, Naseri R, et al. Oxidative stress parameters and Keap 1 variants in T2DM: association with T2DM, diabetic neuropathy, diabetic retinopathy, and obesity. J Clin Lab Anal. 2022;36:e24163. 34861061 10.1002/jcla.24163 PMC8761405

[4] Chen Y, Fu J, Wang Y, Zhang Y, Shi M, Wang C, et al. Association between triglyceride glucose index and subclinical left ventricular systolic dysfunction in patients with type 2 diabetes. Lipids Health Dis. 2023;22:35. 36890516 10.1186/s12944-023-01796-1 PMC9993628

[5] Magliano DJ, Boyko EJ. IDF Diabetes Atlas 10th edition scientific committee. IDF diabetes atlas [Internet]. Brussels: International Diabetes Federation; 2021. http://www.diabetesatlas.org. Accessed 2024 Sep 29.

[6] Hou K, Wu ZX, Chen XY, Wang JQ, Zhang D, Xiao C, et al. Microbiota in health and diseases. Signal Transduct Target Ther. 2022;7:135. 35461318 10.1038/s41392-022-00974-4 PMC9034083

[7] Guo X, Okpara ES, Hu W, Yan C, Wang Y, Liang Q, et al. Interactive relationships between intestinal flora and bile acids. Int J Mol Sci. 2022;23:8343. 35955473 10.3390/ijms23158343 PMC9368770

[8] Zhou Z, Sun B, Yu D, Zhu C. Gut microbiota: an important player in type 2 diabetes mellitus. Front Cell Infect Microbiol. 2022;12:834485. 35242721 10.3389/fcimb.2022.834485 PMC8886906

[9] Schernthaner G, Schernthaner GH. The right place for metformin today. Diabetes Res Clin Pract. 2020;159:107946. 31778746 10.1016/j.diabres.2019.107946

[10] Ríos JL, Francini F, Schinella GR. Natural products for the treatment of type 2 diabetes mellitus. Planta Med. 2015;81:975–94. 26132858 10.1055/s-0035-1546131

[11] Liu Y, Sun M, Yao H, Liu Y, Gao R. Herbal medicine for the treatment of obesity: an overview of scientific evidence from 2007 to 2017. Evid Based Complement Alternat Med. 2017;2017:8943059. 29234439 10.1155/2017/8943059 PMC5632873

[12] Hu X, Wang M, Cai F, Liu L, Cheng Z, Zhao J, et al. A comprehensive review of medicinal Toxicodendron (Anacardiaceae): botany, traditional uses, phytochemistry and pharmacology. J Ethnopharmacol. 2024;318:116829. 37429501 10.1016/j.jep.2023.116829

[13] Liu L, Cai F, Lu Y, Xie Y, Li H, Long C. Comparative lipidomic and metabolomic analyses reveal the mystery of lacquer oil from *Toxicodendron vernicifluum* for the treatment of “Yuezi” disease in Nujiang, China: from anti-inflammation and anti-postpartum depression perspective. Front Pharmacol. 2022;13:914951. 35770099 10.3389/fphar.2022.914951 PMC9234167

[14] Liu X, Cai S, Yi J, Chu C. Chinese sumac fruits (*Rhus chinesis* Mill.) alleviate type 2 diabetes in C57BL/6 mice through repairing islet cell functions, regulating IRS-1/PI3K/AKT pathways and promoting the entry of Nrf2 into the nucleus. Nutrients. 2023;15:4080. 37764863 10.3390/nu15184080 PMC10535436

[15] Alsamri H, Athamneh K, Pintus G, Eid AH, Iratni R. Pharmacological and antioxidant activities of *Rhus coriaria* L. (Sumac). Antioxidants. 2021;10:73. 33430013 10.3390/antiox10010073 PMC7828031

[16] Xu X, Liang T, Lin X, Wen Q, Liang X, Li W, et al. Effect of the total extract of Averrhoa carambola (Oxalidaceae) root on the expression levels of TLR4 and NF-κB in streptozotocin-induced diabetic mice. Cell Physiol Biochem. 2015;36:2307–16. 26279435 10.1159/000430194

[17] Dowman GT, Kleiner DE. Histopathology of nonalcoholic fatty liver disease and nonalcoholic steatohepatitis. Metabolism. 2016;65:1080–6. 26775559 10.1016/j.metabol.2015.11.008 PMC4889547

[18] Ge X, He X, Liu J, Zeng F, Chen L, Xu W, et al. Amelioration of type 2 diabetes by the novel 6, 8-guanidyl luteolin quinone-chromium coordination via biochemical mechanisms and gut microbiota interaction. J Adv Res. 2023;46:173–88. 35700921 10.1016/j.jare.2022.06.003 PMC10105086

[19] Li J, Zhang H, Ouyang H, Xu W, Sun Y, Zhong Y, et al. *Pueraria thomsonii* radix water extract alleviate type 2 diabetes mellitus in db/db mice through comprehensive regulation of metabolism and gut microbiota. Molecules. 2023;28:7471. 38005193 10.3390/molecules28227471 PMC10673130

[20] Liu L, Xie B, Fan M, Candas-Green D, Jiang JX, Wei R, et al. Low-level saturated fatty acid palmitate benefits liver cells by boosting mitochondrial metabolism via CDK1-SIRT3-CPT2 cascade. Dev Cell. 2020;52:196-209.e9. 31866205 10.1016/j.devcel.2019.11.012 PMC6996588

[21] Zhu S, He Y, Lei JN, Liu YF, Xu YJ. The chemical and biological characteristics of fatty acid esters of hydroxyl fatty acids. Nutr Rev. 2025;83:e427–42. 38412339 10.1093/nutrit/nuae005

[22] Al-Sadi RM, Ma TY. IL-1beta causes an increase in intestinal epithelial tight junction permeability. J Immunol. 2007;178:4641–9. 17372023 10.4049/jimmunol.178.7.4641 PMC3724221

[23] Ochoa-Morales PD, González-Ortiz M, Martínez-Abundis E, Pérez-Rubio KG, Patiño-Laguna ADJ. Anti-hyperglycemic effects of propolis or metformin in type 2 diabetes mellitus. Int J Vitam Nutr Res. 2023;93:498–506. 35965421 10.1024/0300-9831/a000760

[24] Xiao S, Fei N, Pang X, Shen J, Wang L, Zhang B, et al. A gut microbiota-targeted dietary intervention for amelioration of chronic inflammation underlying metabolic syndrome. FEMS Microbiol Ecol. 2014;87:357–67. 24117923 10.1111/1574-6941.12228 PMC4255291

[25] Attaye I, Warmbrunn MV, Boot ANAF, van der Wolk SC, Hutten BA, Daams JG, et al. A systematic review and meta-analysis of dietary interventions modulating gut microbiota and cardiometabolic diseases-striving for new standards in microbiome studies. Gastroenterology. 2022;162:1911–32. 35151697 10.1053/j.gastro.2022.02.011

[26] Wen X, Feng X, Xin F, An R, Huang H, Mao L, et al. *B. vulgatus* ameliorates high-fat diet-induced obesity through modulating intestinal serotonin synthesis and lipid absorption in mice. Gut Microbes. 2024;16:2423040. 39569932 10.1080/19490976.2024.2423040 PMC11583587

[27] O’Mahoney LL, Matu J, Price OJ, Birch KM, Ajjan RA, Farrar D, et al. Omega-3 polyunsaturated fatty acids favourably modulate cardiometabolic biomarkers in type 2 diabetes: a meta-analysis and meta-regression of randomized controlled trials. Cardiovasc Diabetol. 2018;17:98. 29981570 10.1186/s12933-018-0740-x PMC6035402

[28] Wang F, Liu HC, Liu XS, Dong SN, Pan D, Yang LG, et al. Effects of ω-3 polyunsaturated fatty acids from different sources on glucolipid metabolism in type 2 diabetic patients with dyslipidemia. Zhonghua Yu Fang Yi Xue Za Zhi. 2019;53:570–5. 31177752 10.3760/cma.j.issn.0253-9624.2019.06.006

[29] Zou J, Xiang Q, Tan D, Shi L, Liu X, Wu Y, et al. Zuogui-jiangtang-qinggan-fang alleviates high-fat diet-induced type 2 diabetes mellitus with non-alcoholic fatty liver disease by modulating gut microbiome-metabolites-short chain fatty acid composition. Biomed Pharmacother. 2023;157:114002. 36410120 10.1016/j.biopha.2022.114002

[30] Wang X, Long D, Hu X, Guo N. Gentiopicroside modulates glucose homeostasis in high-fat-diet and streptozotocin-induced type 2 diabetic mice. Front Pharmacol. 2023;14:1172360. 37601073 10.3389/fphar.2023.1172360 PMC10438990

[31] Shin NR, Whon TW, Bae JW. Proteobacteria: microbial signature of dysbiosis in gut microbiota. Trends Biotechnol. 2015;33:496–503. 26210164 10.1016/j.tibtech.2015.06.011

[32] Dao MC, Everard A, Aron-Wisnewsky J, Sokolovska N, Prifti E, Verger EO, et al. Akkermansia muciniphila and improved metabolic health during a dietary intervention in obesity: relationship with gut microbiome richness and ecology. Gut. 2016;65:426–36. 26100928 10.1136/gutjnl-2014-308778

[33] Song H, Xue H, Zhang Z, Wang J, Li A, Zhang J, et al. Amelioration of type 2 diabetes using four strains of *Lactobacillus* probiotics: effects on gut microbiota reconstitution-mediated regulation of glucose homeostasis, inflammation, and oxidative stress in mice. J Agric Food Chem. 2023;71:20801–14. 37991826 10.1021/acs.jafc.3c04665

[34] Jiang S, Liu A, Ma W, Liu X, Luo P, Zhan M, et al. *Lactobacillus gasseri* CKCC1913 mediated modulation of the gut-liver axis alleviated insulin resistance and liver damage induced by type 2 diabetes. Food Funct. 2023;14:8504–20. 37655696 10.1039/d3fo01701j

[35] Wang K, Liao M, Zhou N, Bao L, Ma K, Zheng Z, et al. Parabacteroides distasonis alleviates obesity and metabolic dysfunctions via production of succinate and secondary bile acids. Cell Rep. 2019;26:222–e355. 30605678 10.1016/j.celrep.2018.12.028

[36] Cui S, Zhao N, Lu W, Zhao F, Zheng S, Wang W, et al. Effect of different *Lactobacillus* species on volatile and nonvolatile flavor compounds in juices fermentation. Food Sci Nutr. 2019;7:2214–23. 31367350 10.1002/fsn3.1010 PMC6657747

[37] Kim DH, Kim SH, Kim SA, Kwak MJ, Han NS, Lee CH. Pathway and production differences in branched-chain hydroxy acids as bioactive metabolites in *Limosilactobacillus fermentum*, *Ligilactobacillus salivarius*, and *Latilactobacillus sakei*. Int J Mol Sci. 2024;25:10112. 39337595 10.3390/ijms251810112 PMC11432647

[38] Mederle AL, Dima M, Stoicescu ER, Căpăstraru BF, Levai CM, Hațegan OA, et al. Impact of gut microbiome interventions on glucose and lipid metabolism in metabolic diseases: a systematic review and meta-analysis. Life (Basel). 2024;14:1485. 39598283 10.3390/life14111485 PMC11595434

[39] Kumar R, Kane H, Wang Q, Hibberd A, Jensen HM, Kim HS, et al. Identification and characterization of a novel species of genus Akkermansia with metabolic health effects in a diet-induced obesity mouse model. Cells. 2022;11:2084. 35805168 10.3390/cells11132084 PMC9265676

[40] Han Y, Ling Q, Wu L, Wang X, Wang Z, Chen J, et al. *Akkermansia muciniphila* inhibits nonalcoholic steatohepatitis by orchestrating TLR2-activated γδT17 cell and macrophage polarization. Gut Microbes. 2023;15:2221485. 37345844 10.1080/19490976.2023.2221485 PMC10288935

[41] Zhang J, Ni Y, Qian L, Fang Q, Zheng T, Zhang M, et al. Decreased abundance of Akkermansia muciniphila leads to the impairment of insulin secretion and glucose homeostasis in lean type 2 diabetes. Adv Sci (Weinh). 2021;8:e2100536. 34085773 10.1002/advs.202100536 PMC8373164

[42] Rao Y, Kuang Z, Li C, Guo S, Xu Y, Zhao D, et al. Gut *Akkermansia muciniphila* ameliorates metabolic dysfunction-associated fatty liver disease by regulating the metabolism of L-aspartate via gut-liver axis. Gut Microbes. 2021;13:1–19. 34030573 10.1080/19490976.2021.1927633 PMC8158032

[43] Özcan E, Seven M, Şirin B, Çakır T, Nikerel E, Teusink B, et al. Dynamic co-culture metabolic models reveal the fermentation dynamics, metabolic capacities and interplays of cheese starter cultures. Biotechnol Bioeng. 2021;118:223–37. 32926401 10.1002/bit.27565 PMC7971941

[44] Zampieri G, Efthimiou G, Angione C. Multi-dimensional experimental and computational exploration of metabolism pinpoints complex probiotic interactions. Metab Eng. 2023;76:120–32. 36720400 10.1016/j.ymben.2023.01.008

[45] de Groot P, Scheithauer T, Bakker GJ, Prodan A, Levin E, Khan MT, et al. Donor metabolic characteristics drive effects of faecal microbiota transplantation on recipient insulin sensitivity, energy expenditure and intestinal transit time. Gut. 2020;69:502–12. 31147381 10.1136/gutjnl-2019-318320 PMC7034343

[46] Kootte RS, Levin E, Salojärvi J, Smits LP, Hartstra AV, Udayappan SD et al. Improvement of insulin sensitivity after lean donor feces in metabolic syndrome is driven by baseline intestinal microbiota composition. Cell Metab. 2017;26:611-9.e6. 10.1016/j.cmet.2017.09.008 28978426

