# Supplementary material for: Effects of Japan tallow on gut microbiota in type 2 diabetic mice
Source: Nutr Metab (Lond). 2026 Jan 11;23:19. doi: 10.1186/s12986-026-01079-3 (PMC12879351; doi:10.1186/s12986-026-01079-3)
Supplement: Supplementary file 1 — Supplementary Material 1 [file 12986_2026_1079_MOESM1_ESM.pdf]

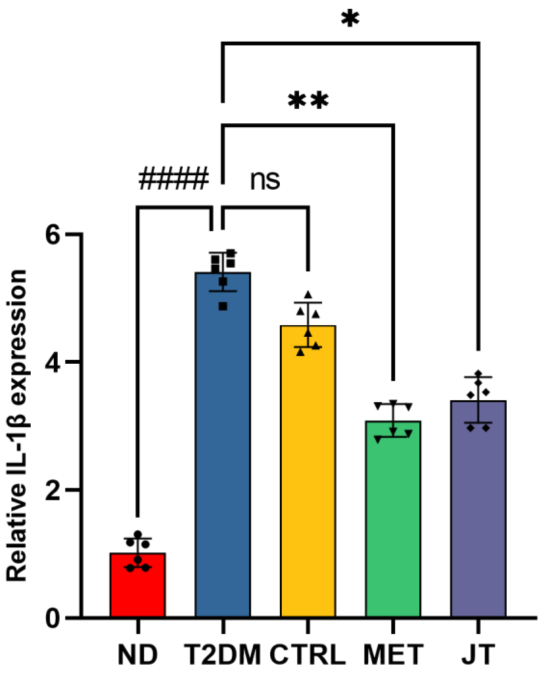

Supplementary Figure 1. JT intervention significantly alters the mRNA expression level of IL-1β. The relative mRNA expression level of IL-1β was determined by RT-qPCR and normalized to GAPDH. Data were expressed as the mean ± SD. n= 6. ####$p < 0.0001$ vs. ND, *$p < 0.05$ vs. T2DM, **$p < 0.01$ vs. T2DM, ns = no significant difference.
